# Contemporary management and outcomes of penetrating traumatic AAST–OIS grade III and IV kidney injuries undergoing laparotomy: a Trauma Quality Improvement Program analysis

**DOI:** 10.1038/s41598-026-47007-6

**Published:** 2026-04-25

**Authors:** Micaela Gomez, Maximilian Peter Forssten, Lucas P. Neff, Lovisa Ekestubbe, Yousef AlHussaini, Babak Sarani, Shahin Mohseni

**Affiliations:** 1 Department of Vascular Surgery, Wake Forest University, Winston-Salem, NC USA; 2 Department of General Surgery, University of Arizona, Tucson, AZ USA; 3 Department of Orthopedic Surgery, Västmanland Hospital Västerås, Västerås, Sweden; 4 School of Medical Sciences, Örebro University, Örebro, Sweden; 5 Department of Pediatric Surgery, Wake Forest School of Medicine, Winston-Salem, NC USA; 6 Department of Orthopedic Surgery, Örebro University Hospital, Örebro, Sweden; 7 Department of Surgery, New Jahra Hospital, Al Jahra, Kuwait; 8 Center of Trauma and Critical Care, George Washington University, Washington, DC USA

**Keywords:** Kidney, Penetrating wounds, Renal salvage, Nephrectomy, Diseases, Medical research, Nephrology, Urology

## Abstract

This study aimed to compare the outcomes of renal salvage (no nephrectomy) to nephrectomy patients with a penetrating American Association for the Surgery of Trauma–Organ Injury Scale grade III or IV kidney injury. The 2013–2021 Trauma Quality Improvement Program dataset was queried for patients with a penetrating grade III or IV kidney injury who underwent laparotomy within 24 h of admission. The association between nephrectomy and binary outcomes was investigated using Poisson regression models, with results presented as a prevalence ratio (PR) and corresponding 95% confidence interval (CI). A total of 2,214 grade III and 2,669 grade IV kidney injuries were identified. 89% of grade III injuries and 54% of grade IV injuries were managed without nephrectomy. After adjustment for confounding, nephrectomy in patients with a grade III penetrating kidney injury was associated with an increased risk of mortality [adjusted PR (95% CI): 1.53 (1.06–2.20), *p* = 0.023], complications [adjusted PR (95% CI): 1.25 (1.05–1.48), *p* = 0.010], post-complication mortality [adjusted PR (95% CI): 1.80 (1.09–2.96), *p* = 0.022], and ICU admission [adjusted PR (95% CI): 1.05 (1.00-1.11), *p* = 0.046], compared to renal salvage. In patients with a grade IV penetrating kidney injury, nephrectomy was only associated with an increased risk of ICU admission [adjusted PR (95% CI): 1.06 (1.02–1.09), *p* = 0.001], compared to renal salvage. Consequently, in patients with a grade III penetrating kidney injury, nephrectomy was associated with an increased risk of adverse outcomes. Among patients with a grade IV injury, nephrectomy was associated only with an increased risk of ICU admission. However, given the potential for residual confounding, these findings should be interpreted with caution.

**Supplementary Information:**

The online version contains supplementary material available at 10.1038/s41598-026-47007-6.

## Introduction

The kidney is the most commonly injured organ of the genitourinary system following trauma^1,2^. Most renal trauma is the result of blunt mechanisms, while penetrating injuries make up only 10–20% of total injuries depending on the population being studied^3–5^. Over the last several decades, the treatment strategies for renal trauma have evolved significantly, shifting from operative management (OM) to non-operative management (NOM)^6^. Non-operative management is now the mainstay practice for nearly all blunt renal trauma; however, there have also been favorable outcomes reported in select penetrating injuries^7–13^.

The management of penetrating renal trauma, however, is more nuanced as compared to blunt renal trauma. For example, penetrating renal trauma has a higher nephrectomy rate per grade of injury, higher rate of concomitant intraabdominal organs injured, and higher failure rate of angioembolization compared to blunt trauma^14–16^. Select patients can be managed nonoperatively based on radiographic findings including severity of renal injury, lack of evidence of ongoing bleeding, and hemodynamic parameters, which makes the decision to empirically explore all zone II retroperitoneal hematomas following penetrating trauma difficult^6,17^.

Routine retroperitoneal exploration of penetrating zone II hematomas is associated with nephrectomy for AAST grade III and higher renal injuries^18^. The incidence of nephrectomy is as high as 64% when the retroperitoneum is explored in these patients^18^. With increasing reports of success of high-grade penetrating renal injuries managed non-operatively, we sought to elucidate the comparative outcomes of patients with an American Association for the Surgery of Trauma–Organ Injury Scale Grade III or IV penetrating kidney injury, who underwent laparotomy within 24 h of admission, based on whether they underwent nephrectomy (total or partial nephrectomy) versus renal salvage (no nephrectomy). We hypothesized that renal salvage, as a surrogate for not exploring the retroperitoneum, may be feasible in patients with high-grade renal injuries without compromising outcomes.

## Methods

The current analysis was granted exemption from the institutional review board’s ethical approval due to the use of an anonymized, retrospective dataset for all analyses. All aspects of the current investigation were performed in accordance with the Declaration of Helsinki, the Strengthening the Reporting of Observational Studies in Epidemiology (STROBE), and the Reporting of Studies Conducted Using Observational Routinely-Collected Health Data (RECORD) guidelines^19^.

Data for the current analysis were obtained from the American College of Surgeons Trauma Quality Improvement Program (TQIP) dataset. This included information pertaining to patient demographics, clinical characteristics, management strategy, and in-hospital outcomes. All adult patients (≥ 18 years old) registered in TQIP between 2013 and 2021 who suffered a grade III or IV kidney injury due to penetrating trauma and underwent a laparotomy within 24 h were considered for inclusion. Patients were excluded if they had an abbreviated injury scale (AIS) of 6 in any region since these injuries are generally not survivable. All codes used to define injuries and procedures are included in Supplemental Table 1. Releasing the tamponade provided by the intact retroperitoneal sheath during trauma laparotomy may increase bleeding and often results in nephrectomy. Renal salvage may therefore be interpreted as a surrogate for not exploring the retroperitoneum when limiting the analysis to patients who underwent laparotomy; however, TQIP cannot explicitly distinguish between exploration with renal salvage from no exploration at all.

### Statistical analysis

Patients were grouped based on the management strategy used for managing the kidney injury: nephrectomy (total or partial nephrectomy) versus renal salvage (no nephrectomy). Grade III and IV kidney injuries were analyzed separately. Continuous variables were described using medians and interquartile ranges (IQRs) due to their non-normal distribution. Subsequently, the Mann-Whitney U-test was employed to assess the statistical significance of differences between the treatment groups. Categorical variables, on the other hand, were presented as counts and percentages. To evaluate the statistical significance of differences either the Chi-squared test or Fisher’s exact test was utilized. Outcomes included in-hospital mortality, complications (myocardial infarction, cardiac arrest with CPR, stroke, acute kidney injury, acute respiratory distress syndrome, deep vein thrombosis, pulmonary embolism, urinary tract infection, pneumonia, surgical site infection, sepsis, decubitus ulcer, unplanned intubation, unplanned admission to the OR, and unplanned admission to the ICU), any complication (excluding acute kidney injury), post-complication mortality, ICU admission, and hospital length of stay (LOS). Post-complication mortality was defined as mortality following any in-hospital complication.

In order to adjust for confounding, Poisson regression models with robust standard errors were utilized when the outcome was mortality, complications, post-complication mortality, or ICU admission. Quantile regression models were employed with the same purpose when the outcome was LOS. Predictors in all models included management strategy (renal salvage or nephrectomy) age, sex, race, highest AIS in each region, vitals on admission, presence of renal artery or renal vein injury, comorbidities (hypertension, previous myocardial infarction, congestive heart failure, history of peripheral vascular disease, cerebrovascular disease, diabetes mellitus, chronic kidney disease, dementia, functionally dependent health status, chronic obstructive pulmonary disease, smoking status, cirrhosis, coagulopathy, drug use disorder, alcohol use disorder, major psychiatric illness), advanced directives limiting care, surgery on the gastrointestinal system, surgery on the hepatobiliary system or pancreas, surgery on the urinary system (excluding nephrectomy), units of packed red blood cells transfused within 4 h, mechanism of injury (gunshot wound or other penetrating trauma), trauma center level, and year of admission. Results of the Poisson regression models are presented as an adjusted prevalence ratio (PR) with corresponding 95% confidence intervals (CIs). Results of the quantile regression models are presented as the change in median LOS with corresponding 95% CIs. A subgroup analysis was also performed on patients with a systolic blood pressure ≥ 90 mmHg on admission.

Statistical significance was defined as a two-sided p-value < 0.05. Missing data were managed using multiple imputation by chained Eq. 14 imputed datasets were generated in parallel over 10 iterations using classification and regression trees. The imputation model incorporated all outcomes, predictors, and potential confounders listed above. Analyses were performed on each imputed dataset individually and subsequently pooled using Rubin’s Rules in order to also account for variability between the imputed datasets. Analyses were performed using the statistical programming language R version 4.2.2 (R Foundation for Statistical Computing, Vienna, Austria) using the *tidyverse*, *parallel*, *quantreg*, *sandwich*, and *mice*packages^20^.

## Results

**Fig. 1:**
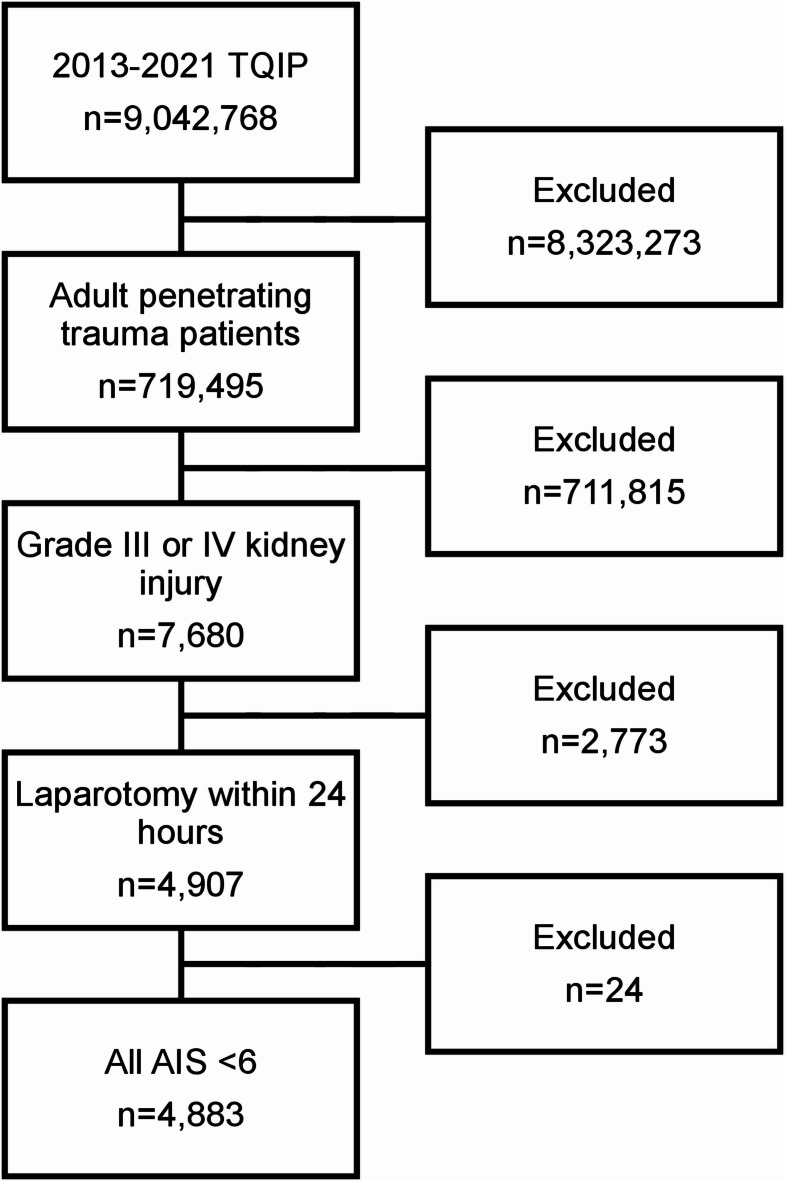
Flow chart.

A total of 2,214 patients with a grade III and 2,669 patients with a grade IV kidney injury were included after applying the inclusion and exclusion criteria (Fig. 1). 86 Patients (*N* = 1.8%) had an AIS code for both a grade III and a grade IV kidney injury, these patients were only included in the analysis of patients with a grade IV kidney injury. The majority of grade III injuries were managed without nephrectomy (89%, *N* = 1,965) while about half of grade IV injuries were managed without nephrectomy (54%, *N* = 1,439). There were no statistically significant differences in age or sex between those managed with and without nephrectomy (Table 1).

**Table 1:** Demographics of patients with a grade III or IV kidney injury due to penetrating trauma.

	Grade III			Grade IV		
	Renal Salvage (*N* = 1,965)	Nephrectomy (*N* = 249)	*P*-value	Renal Salvage (*N* = 1,439)	Nephrectomy (*N* = 1,230)	*P*-value
Age, median [IQR]	29 [23–38]	30 [24–39]	0.375	29 [23–37]	29 [23–38]	0.485
Sex, n (%)			0.786			0.930
Female	229 (11.7)	27 (10.8)		151 (10.5)	127 (10.3)	
Male	1,728 (87.9)	221 (88.8)		1,283 (89.2)	1,100 (89.4)	
Missing	8 (0.4)	1 (0.4)		5 (0.3)	3 (0.2)	
Race, n (%)						
White	536 (27.3)	74 (29.7)	0.476	389 (27.0)	326 (26.5)	0.841
Black	1,119 (56.9)	131 (52.6)	0.200	833 (57.9)	708 (57.6)	0.993
Asian	12 (0.6)	5 (2.0)	0.035	12 (0.8)	10 (0.8)	1.000
American Indian	25 (1.3)	3 (1.2)	1.00	13 (0.9)	11 (0.9)	1.000
Pacific islander	4 (0.2)	1 (0.4)	0.450	2 (0.1)	3 (0.2)	0.666
Other	214 (10.9)	30 (12.0)	0.669	139 (9.7)	132 (10.7)	0.376
Missing	13 (0.7)	1 (0.4)		20 (1.4)	22 (1.8)	
Hypertension, n (%)	146 (7.4)	22 (8.8)	0.508	99 (6.9)	93 (7.6)	0.546
Previous myocardial infarction, n (%)	0 (0.0)	0 (0.0)	NA	3 (0.2)	2 (0.2)	1.00
Congestive heart failure, n (%)	8 (0.4)	1 (0.4)	1.00	2 (0.1)	6 (0.5)	0.154
History of peripheral vascular disease, n (%)	2 (0.1)	0 (0.0)	1.00	1 (0.1)	0 (0.0)	1.00
Cerebrovascular disease, n (%)	2 (0.1)	0 (0.0)	1.00	3 (0.2)	7 (0.6)	0.202
Diabetes mellitus, n (%)	84 (4.3)	9 (3.6)	0.748	51 (3.5)	34 (2.8)	0.302
Chronic renal failure, n (%)	2 (0.1)	1 (0.4)	0.301	1 (0.1)	3 (0.2)	0.340
Dementia, n (%)	1 (0.1)	0 (0.0)	1.00	0 (0.0)	1 (0.1)	0.461
Functionally dependent health status, n (%)	9 (0.5)	0 (0.0)	0.610	2 (0.1)	5 (0.4)	0.259
COPD, n (%)	40 (2.0)	7 (2.8)	0.571	30 (2.1)	25 (2.0)	1.000
Current smoker, n (%)	570 (29.0)	51 (20.5)	0.006	412 (28.6)	274 (22.3)	< 0.001
Cirrhosis, n (%)	4 (0.2)	2 (0.8)	0.139	4 (0.3)	4 (0.3)	1.00
Major psychiatric illness, n (%)	172 (8.8)	22 (8.8)	1.000	109 (7.6)	99 (8.0)	0.702
Coagulopathy, n (%)	9 (0.5)	0 (0.0)	0.610	13 (0.9)	14 (1.1)	0.682
Currently receiving chemotherapy for cancer, n (%)	1 (0.1)	1 (0.4)	0.212	1 (0.1)	0 (0.0)	1.00
Disseminated cancer, n (%)	1 (0.1)	1 (0.4)	0.212	0 (0.0)	1 (0.1)	0.461
Drug use disorder, n (%)	320 (16.3)	32 (12.9)	0.192	239 (16.6)	145 (11.8)	< 0.001
Alcohol use disorder, n (%)	122 (6.2)	11 (4.4)	0.328	74 (5.1)	70 (5.7)	0.590
Advanced directive limiting care, n (%)	5 (0.3)	0 (0.0)	1.00	2 (0.1)	5 (0.4)	0.259

*COPD* chronic obstructive pulmonary disease.

For patients with a grade III injury, those undergoing nephrectomy had a higher median Injury Severity Score (ISS) compared to those who underwent renal salvage [20 (16–32) vs. 19 (13–27); *p* = 0.005]. Patients with a grade IV renal injury had similar median ISS [25 (17–33) vs. 25 (17–33) *p* = 0.370], but those who underwent renal salvage were more likely to have a higher AIS in the neck, spine, thorax, upper extremity, and lower extremity. Additionally, patients who underwent nephrectomy were more likely to be hypotensive at presentation in both grade III [22.5% vs. 15.8%; *p* = 0.007] and grade IV injury cohorts [23.5% vs. 17.5%; *p* < 0.001] and to have lower presenting GCS (Table 2).

**Table 2:** Clinical characteristics of patients with a grade III or IV kidney injury due to penetrating trauma.

	Grade III			Grade IV		
	Renal Salvage (*N* = 1,965)	Nephrectomy (*N* = 249)	*P*-value	Renal Salvage (*N* = 1,439)	Nephrectomy (*N* = 1,230)	*P*-value
ISS, median [IQR]	19 [13–27]	20 [16–32]	0.005	25 [17–33]	25 [17–33]	0.370
Head AIS, n (%)			0.307			0.860
Injury not present	1,860 (94.7)	240 (96.4)		1,372 (95.3)	1,167 (94.9)	
1	60 (3.1)	4 (1.6)		31 (2.2)	27 (2.2)	
2	13 (0.7)	0 (0.0)		8 (0.6)	7 (0.6)	
3	18 (0.9)	3 (1.2)		12 (0.8)	16 (1.3)	
4	8 (0.4)	0 (0.0)		7 (0.5)	4 (0.3)	
5	6 (0.3)	2 (0.8)		8 (0.6)	8 (0.7)	
Missing	0 (0.0)	0 (0.0)		1 (0.1)	1 (0.1)	
Face AIS, n (%)			0.580			0.065
Injury not present	1,795 (91.3)	235 (94.4)		1,323 (91.9)	1,160 (94.3)	
1	122 (6.2)	10 (4.0)		71 (4.9)	42 (3.4)	
2	43 (2.2)	4 (1.6)		43 (3.0)	26 (2.1)	
3	3 (0.2)	0 (0.0)		0 (0.0)	1 (0.1)	
4	1 (0.1)	0 (0.0)		2 (0.1)	1 (0.1)	
Missing	1 (0.1)	0 (0.0)		0 (0.0)	0 (0.0)	
Neck AIS, n (%)			0.615			0.015
Injury not present	1,898 (96.6)	243 (97.6)		1,382 (96.0)	1,209 (98.3)	
1	42 (2.1)	5 (2.0)		37 (2.6)	14 (1.1)	
2	16 (0.8)	0 (0.0)		8 (0.6)	3 (0.2)	
3	5 (0.3)	1 (0.4)		8 (0.6)	3 (0.2)	
4	4 (0.2)	0 (0.0)		3 (0.2)	1 (0.1)	
5	0 (0.0)	0 (0.0)		1 (0.1)	0 (0.0)	
Spine AIS, n (%)			0.775			0.020
Injury not present	1,423 (72.4)	186 (74.7)		1,005 (69.8)	906 (73.7)	
2	365 (18.6)	40 (16.1)		305 (21.2)	222 (18.0)	
3	40 (2.0)	5 (2.0)		30 (2.1)	28 (2.3)	
4	28 (1.4)	5 (2.0)		24 (1.7)	7 (0.6)	
5	109 (5.5)	12 (4.8)		74 (5.1)	62 (5.0)	
Missing	0 (0.0)	1 (0.4)		1 (0.1)	5 (0.4)	
Thorax AIS, n (%)			0.699			0.021
Injury not present	708 (36.0)	95 (38.2)		529 (36.8)	521 (42.4)	
1	176 (9.0)	18 (7.2)		108 (7.5)	82 (6.7)	
2	230 (11.7)	28 (11.2)		173 (12.0)	134 (10.9)	
3	613 (31.2)	81 (32.5)		436 (30.3)	317 (25.8)	
4	199 (10.1)	20 (8.0)		160 (11.1)	153 (12.4)	
5	38 (1.9)	7 (2.8)		33 (2.3)	22 (1.8)	
Missing	0 (0.0)	0 (0.0)		0 (0.0)	1 (0.1)	
Abdomen AIS, n (%)			< 0.001			< 0.001
3	1,263 (64.3)	126 (50.6)		0 (0.0)	0 (0.0)	
4	505 (25.7)	71 (28.5)		1,198 (83.3)	875 (71.1)	
5	197 (10.0)	52 (20.9)		241 (16.7)	355 (28.9)	
Upper extremity AIS, n (%)			0.728			0.001
Injury not present	1,352 (68.8)	176 (70.7)		1,002 (69.6)	941 (76.5)	
1	335 (17.0)	42 (16.9)		222 (15.4)	138 (11.2)	
2	177 (9.0)	17 (6.8)		134 (9.3)	94 (7.6)	
3	100 (5.1)	14 (5.6)		78 (5.4)	56 (4.6)	
4	1 (0.1)	0 (0.0)		2 (0.1)	0 (0.0)	
Missing	0 (0.0)	0 (0.0)		1 (0.1)	1 (0.1)	
Lower extremity AIS, n (%)			0.957			0.019
Injury not present	1,491 (75.9)	186 (74.7)		1,062 (73.8)	956 (77.7)	
1	223 (11.3)	31 (12.4)		170 (11.8)	122 (9.9)	
2	65 (3.3)	7 (2.8)		51 (3.5)	50 (4.1)	
3	168 (8.5)	23 (9.2)		146 (10.1)	90 (7.3)	
4	15 (0.8)	2 (0.8)		7 (0.5)	11 (0.9)	
5	1 (0.1)	0 (0.0)		1 (0.1)	0 (0.0)	
Missing	2 (0.1)	0 (0.0)		2 (0.1)	1 (0.1)	
External/Other AIS, n (%)			0.511			0.651
Injury not present	1,880 (95.7)	241 (96.8)		1,367 (95.0)	1,174 (95.4)	
1	85 (4.3)	8 (3.2)		72 (5.0)	56 (4.6)	
Systolic blood pressure < 90 mmHg, n (%)	311 (15.8)	56 (22.5)	0.007	252 (17.5)	289 (23.5)	< 0.001
Missing	48 (2.4)	10 (4.0)		42 (2.9)	48 (3.9)	
Pulse > 100, n (%)	819 (41.7)	122 (49.0)	0.048	650 (45.2)	567 (46.1)	0.597
Missing	32 (1.6)	1 (0.4)		29 (2.0)	29 (2.4)	
Temperature < 35 °C, n (%)	50 (2.5)	12 (4.8)	0.043	46 (3.2)	46 (3.7)	0.266
Missing	504 (25.6)	73 (29.3)		444 (30.9)	453 (36.8)	
Temperature ≥ 38 °C, n (%)	12 (0.6)	2 (0.8)	0.656	7 (0.5)	4 (0.3)	0.765
Missing	504 (25.6)	73 (29.3)		444 (30.9)	453 (36.8)	
Saturation < 90%, n (%)	67 (3.4)	13 (5.2)	0.202	60 (4.2)	61 (5.0)	0.288
Missing	104 (5.3)	14 (5.6)		120 (8.3)	137 (11.1)	
Respiratory rate > 20, n (%)	850 (43.3)	118 (47.4)	0.168	621 (43.2)	533 (43.3)	0.570
Missing	77 (3.9)	13 (5.2)		62 (4.3)	79 (6.4)	
Respiratory rate < 12, n (%)	46 (2.3)	13 (5.2)	0.013	56 (3.9)	72 (5.9)	0.016
Missing	77 (3.9)	13 (5.2)		62 (4.3)	79 (6.4)	
Glasgow Coma Scale, n (%)			< 0.001			0.003
Mild (14–15)	1,606 (81.7)	182 (73.1)		1,103 (76.7)	872 (70.9)	
Moderate (9–13)	141 (7.2)	20 (8.0)		117 (8.1)	111 (9.0)	
Severe (3–8)	182 (9.3)	46 (18.5)		192 (13.3)	217 (17.6)	
Missing	36 (1.8)	1 (0.4)		27 (1.9)	30 (2.4)	
Renal artery injury, n (%)	23 (1.2)	9 (3.6)	0.007	72 (5.0)	80 (6.5)	0.113
Renal vein injury, n (%)	9 (0.5)	9 (3.6)	< 0.001	37 (2.6)	74 (6.0)	< 0.001
Type of nephrectomy, n (%)			< 0.001			< 0.001
None	1,965 (100.0)	0 (0.0)		1,439 (100.0)	0 (0.0)	
Partial	0 (0.0)	78 (31.3)		0 (0.0)	222 (18.0)	
Total	0 (0.0)	171 (68.7)		0 (0.0)	1,008 (82.0)	
Surgery on the gastrointestinal system, n (%)	1,390 (70.7)	192 (77.1)	0.043	929 (64.6)	944 (76.7)	< 0.001
Surgery on the hepatobiliary system or pancreas, n (%)	753 (38.3)	122 (49.0)	0.001	516 (35.9)	581 (47.2)	< 0.001
Surgery on the urinary system*, n (%)	860 (43.8)	106 (42.6)	0.771	774 (53.8)	421 (34.2)	< 0.001
Units of PRBC transfused within 4 h, median [IQR]	0.06 [0.00–4.8.00.8]	4.8 [0.02–12.02]	< 0.001	1.2 [0.00–5.6.00.6]	3.9 [0.00–12]	< 0.001
Missing, n (%)	0 (0.0)	0 (0.0)		2 (0.1)	3 (0.2)	
Trauma center level, n (%)			0.007			< 0.001
I	1,160 (59.0)	137 (55.0)		822 (57.1)	632 (51.4)	
II	352 (17.9)	32 (12.9)		222 (15.4)	172 (14.0)	
IIII	19 (1.0)	2 (0.8)		19 (1.3)	8 (0.7)	
Not verified	434 (22.1)	78 (31.3)		376 (26.1)	418 (34.0)	
Gunshot wound, n (%)	1,649 (83.9)	216 (86.7)	0.288	1,286 (89.4)	1,144 (93.0)	0.001
Year of admission, n (%)			0.988			< 0.001
2013	93 (4.7)	12 (4.8)		118 (8.2)	159 (12.9)	
2014	126 (6.4)	14 (5.6)		127 (8.8)	152 (12.4)	
2015	136 (6.9)	19 (7.6)		163 (11.3)	190 (15.4)	
2016	214 (10.9)	33 (13.3)		121 (8.4)	108 (8.8)	
2017	215 (10.9)	26 (10.4)		174 (12.1)	108 (8.8)	
2018	228 (11.6)	28 (11.2)		162 (11.3)	99 (8.0)	
2019	252 (12.8)	30 (12.0)		145 (10.1)	113 (9.2)	
2020	369 (18.8)	47 (18.9)		210 (14.6)	151 (12.3)	
2021	332 (16.9)	40 (16.1)		219 (15.2)	150 (12.2)	

*ISS* Injury Severity Score, *AIS* Abbreviated Injury Scale, *PRBC* Packed red blood cells.

*Excluding partial or total nephrectomy.

Crude outcomes are presented in Table 3. After adjusting for potential confounders, nephrectomy among patients with a grade III penetrating kidney injury was associated with a 53% increase in the risk of mortality [adjusted PR (95% CI): 1.53 (1.06–2.20), *p* = 0.023], a 25% increased risk of complications [adjusted PR (95% CI): 1.25 (1.05–1.48), *p* = 0.010], a 21% increased risk of complications, excluding acute kidney injury, [adjusted PR (95% CI): 1.21 (1.01–1.45), *p* = 0.035], an 80% increased risk of post-complication mortality [adjusted PR (95% CI): 1.80 (1.09–2.96), *p* = 0.022], and a 5% increased risk of ICU admission [adjusted PR (95% CI): 1.05 (1.00–1.11), *p* = 0.046], compared to renal salvage. However, no difference was observed in the overall hospital length of stay (Table 4). In the subgroup analysis of patients with a grade III penetrating kidney injury without hypotension on admission, there remained a statistically significant association between nephrectomy and a 24% increased risk of complications [adjusted PR (95% CI): 1.24 (1.01–1.52), *p* = 0.040], a 9% increased risk of ICU admission [adjusted PR (95% CI): 1.09 (1.03–1.16), *p* = 0.004], as well as a median hospital length of stay that was 1.7 days longer [change in median (95% CI): 1.70 (0.02–3.38), *p* = 0.048], compared to renal salvage (Supplemental Table 2).

**Table 3:** Crude outcomes in patients with a grade III or IV kidney injury due to penetrating trauma.

	Grade III			Grade IV		
	Renal Salvage (*N* = 1,965)	Nephrectomy (*N* = 249)	*P*-value	Renal Salvage (*N* = 1,439)	Nephrectomy (*N* = 1,230)	*P*-value
In-hospital mortality, n (%)	119 (6.1)	45 (18.1)	< 0.001	181 (12.6)	206 (16.7)	0.003
Any complication, n (%)	523 (26.6)	104 (41.8)	< 0.001	463 (32.2)	463 (37.6)	0.004
Myocardial infarction	2 (0.1)	1 (0.4)	0.301	5 (0.3)	4 (0.3)	1.00
Cardiac arrest with CPR	64 (3.3)	24 (9.6)	< 0.001	77 (5.4)	85 (6.9)	0.109
Stroke	8 (0.4)	0 (0.0)	0.609	5 (0.3)	2 (0.2)	0.463
Acute kidney injury	78 (4.0)	28 (11.2)	< 0.001	67 (4.7)	136 (11.1)	< 0.001
ARDS	23 (1.2)	7 (2.8)	0.071	35 (2.4)	35 (2.8)	0.586
DVT	76 (3.9)	17 (6.8)	0.043	57 (4.0)	61 (5.0)	0.248
Pulmonary embolism	55 (2.8)	6 (2.4)	0.882	36 (2.5)	29 (2.4)	0.909
Urinary tract infection	43 (2.2)	1 (0.4)	0.054	45 (3.1)	34 (2.8)	0.662
Pneumonia	54 (2.7)	10 (4.0)	0.355	46 (3.2)	56 (4.6)	0.085
Surgical site infection	138 (7.0)	28 (11.2)	0.024	116 (8.1)	122 (9.9)	0.107
Sepsis	60 (3.1)	9 (3.6)	0.775	46 (3.2)	59 (4.8)	0.043
Decubitus ulcer	29 (1.5)	8 (3.2)	0.060	24 (1.7)	29 (2.4)	0.257
Unplanned intubation	64 (3.3)	8 (3.2)	1.000	45 (3.1)	50 (4.1)	0.231
Unplanned admission to the OR	133 (6.8)	23 (9.2)	0.193	118 (8.2)	141 (11.5)	0.006
Unplanned admission to the ICU	95 (4.8)	12 (4.8)	1.00	73 (5.1)	63 (5.1)	1.000
Any complication, excluding acute kidney injury n (%)	502 (25.5)	96 (38.6)	< 0.001	442 (30.7)	436 (35.4)	0.011
Post-complication mortality	66 (3.4)	28 (11.2)	< 0.001	91 (6.3)	91 (7.4)	0.307
ICU admission, n (%)	1,580 (80.4)	215 (86.3)	0.030	1,184 (82.3)	1,065 (86.6)	0.003
Length of stay, median [IQR]	10 [6.2–19]	13 [7.0–25]	0.017	12 [6.7–21]	13 [6.4–23]	0.515
Missing, n (%)	22 (1.1)	4 (1.6)		18 (1.3)	6 (0.5)	

*ARDS* Acute respiratory distress syndrome, *DVT* Deep vein thrombosis.

**Table 4:** Association between nephrectomy and outcomes in patients with a grade III kidney injury due to penetrating trauma.

Outcome	Renal Salvage	Nephrectomy PR (95% CI)	*P*-Value
In-hospital mortality	Reference	1.53 (1.06–2.20)	0.023
Any complication	Reference	1.25 (1.05–1.48)	0.010
Any complication, excluding acute kidney injury	Reference	1.21 (1.01–1.45)	0.035
Post-complication mortality	Reference	1.80 (1.09–2.96)	0.022
ICU admission	Reference	1.05 (1.00–1.11)	0.046
		**Change in median (95% CI)**	**P-Value**
Length of stay	Reference	1.08 (−0.62-2.78)	0.214

PRs are calculated using Poisson regression models with robust standard errors. Median length of stay is calculated using a quantile regression model. Missing values were managed using multiple imputation by chained equations. All analyses were adjusted for age, sex, race, highest abbreviated injury scale in each region, vitals on admission, presence of renal artery or renal vein injury, comorbidities, advanced directives limiting care, surgery on the gastrointestinal system, surgery on the hepatobiliary system or pancreas, surgery on the urinary system (excluding nephrectomy), units of packed red blood cells transfused within 4 h, mechanism of injury, trauma center level, and year of admission.

*PR* Prevalence Ratio, *CI* Confidence Interval.

Undergoing nephrectomy as a result of a grade IV penetrating kidney injury was associated with a 6% increased risk of ICU admission [adjusted PR (95% CI): 1.06 (1.02–1.09), *p* = 0.001] compared to renal salvage after adjusting for confounding. There were no significant differences in the risk of mortality, complications, post-complication mortality, or the overall hospital length of stay (Table 5). In the subgroup analysis of patients with a grade IV penetrating kidney injury without hypotension on admission, there was no statistically significant association between nephrectomy and any of the investigated outcomes, compared to renal salvage (Supplemental Table 3).

**Table 5:** Association between nephrectomy and outcomes in patients with a grade IV kidney injury due to penetrating trauma.

Outcome	Renal Salvage	Nephrectomy PR (95% CI)	*P*-Value
In-hospital mortality	Reference	0.96 (0.80–1.16)	0.673
Any complication	Reference	0.97 (0.87–1.08)	0.586
Any complication, excluding acute kidney injury	Reference	0.96 (0.85–1.07)	0.456
Post-complication mortality	Reference	0.76 (0.55–1.05)	0.099
ICU admission	Reference	1.06 (1.02–1.09)	0.001
		**Change in median (95% CI)**	**P-Value**
Length of stay	Reference	0.29 (−0.72-1.30)	0.572

PRs are calculated using Poisson regression models with robust standard errors. Median length of stay is calculated using a quantile regression model. Missing values were managed using multiple imputation by chained equations. All analyses were adjusted for age, sex, race, highest abbreviated injury scale in each region, vitals on admission, presence of renal artery or renal vein injury, comorbidities, advanced directives limiting care, surgery on the gastrointestinal system, surgery on the hepatobiliary system or pancreas, surgery on the urinary system (excluding nephrectomy), units of packed red blood cells transfused within 4 h, mechanism of injury, trauma center level, and year of admission.

*PR* Prevalence Ratio, *CI* Confidence Interval.

## Discussion

There has been a distinct evolution towards kidney-sparing management of patients with renal injuries, including reports in select penetrating renal trauma^6^. However, it is unclear which treatment strategy is best for this subset of patients, especially those who are already undergoing a laparotomy for concomitant injuries. This study found that the majority of patients with a grade III renal injury (89%) or a grade IV renal injury (54%) who underwent laparotomy for concomitant injury were managed without nephrectomy. After adjusting for confounding, renal salvage was not associated with an increased risk of mortality, complications, post-complication mortality, ICU admission, or longer hospital length of stay in patients with a grade IV penetrating kidney injury. This remained true in the subgroup analysis of patients without hypotension on admission. In patients with a grade III penetrating kidney injury, nephrectomy was instead associated with an increased adjusted risk of mortality, complications, post-complication mortality, and ICU admission, compared to renal salvage. However, this finding should be interpreted with caution given the risk of residual confounding in retrospective analyses.

These results suggest patients with a grade III or IV penetrating renal injury who are already undergoing laparotomy can in select cases be managed without nephrectomy. Extrapolating from this finding, it may further be hypothesized that routine exploration of the retroperitoneum, which increases the risk of subsequent nephrectomy^18^, might not be required in all cases of grade III or IV penetrating renal injury. However, exploration is likely still indicated in patients who manifest signs of ongoing bleeding, such as an expanding hematoma and/or hemodynamic instability^9,21^. This is in part supported by current findings, in which patients who were hypotensive at admission were more likely to undergo nephrectomy.

Of note, our analysis found that patients with a grade III or IV renal injury that were managed without nephrectomy were more commonly treated at Level 1 trauma centers. This trend is likely reflective of the fact that attempting renal salvage in patients with these high-grade renal injuries require extensive resources including continuous monitoring for changes in clinical status and round-the-clock availability of multidisciplinary teams, including interventional radiology and surgeons, that are highly adept in managing these injuries in the case of patient deterioration. Furthermore, this study indicated that patients who underwent a nephrectomy were more likely to be hypotensive and have a lower GCS at time of presentation in both grade III and grade IV renal injuries. Patients with a grade III injury that were managed with a nephrectomy had a higher median ISS and patients with a grade IV renal injury subjected to nephrectomy had similar median ISS but had higher AIS in the abdomen. Collectively, these findings underscore the critical and well-established clinical decision-making process, wherein prompt and definitive intervention, such as nephrectomy, is deemed the safest course of action when dealing with severely injured and unstable trauma patients.

As this study was a retrospective cohort study using a large national database, there are several limitations. Of particular note is the risk of residual confounding inherent to retrospective analyses. Based on the data available in TQIP it was not possible to adjust for the physiologic status beyond admission vitals, intraoperative instability, intraoperative bleeding severity, surgeon judgment of salvageability, or the timing and availability of imaging. Data on long term outcomes such as renal function, dialysis dependence, hypertension, and quality of life are also not recorded in TQIP. Additionally, it is not clear from the available data that the increased risk of complications and mortality can solely be attributed to the nephrectomy, although we attempted to control for this by accounting for severity of injury and adjusting for other surgical procedures/interventions. Patients in the nephrectomy groups were more likely to have additional surgical procedures including surgery on the hepatobiliary, pancreas, gastrointestinal, and urinary systems. These surgical procedures each come with their own complication profiles which could ultimately affect the overall outcome. Another limitation of the study is the ambiguity surrounding the timing of renal injury diagnosis through imaging. It is probable that some of the patients, particularly those who presented with hypotension, were taken immediately to the operating room prior to any imaging. Concern for a renal injury could have arisen in the operating room by identification of a zone II hematoma leading to exploration of the retroperitoneal space and nephrectomy. Furthermore, as length of stay analyses include all patients, the higher early mortality among nephrectomy patients could shorten observed length of stay, and therefore length of stay comparisons may be influenced by differential survival. Overall, the findings of the current investigation suggest that routine nephrectomy in grade III and grade IV kidney injuries should not be practiced by default. However, given the previously stated limitations, further prospective study is required before changes to current clinical practice can be recommended.

## Conclusion

In patients with a grade III penetrating kidney injury, nephrectomy was associated with an increased risk of adverse outcomes. Among patients with a grade IV injury, nephrectomy was associated only with an increased risk of ICU admission. However, given the potential for residual confounding, these findings should be interpreted with caution.

## Supplementary Information

Below is the link to the electronic supplementary material.

Supplementary Material 1

Supplementary Material 2

## Data Availability

The data that support the findings of this study were obtained from the American College of Surgeons Trauma Quality Improvement Program (TQIP) database. Restrictions apply to the availability of these data, which were used under license for the current study, and are therefore not publicly available. Data are, however, available from the corresponding author upon reasonable request and with permission of the American College of Surgeons.
