# Supplementary material for: Contemporary management and outcomes of penetrating traumatic AAST–OIS grade III and IV kidney injuries undergoing laparotomy: a Trauma Quality Improvement Program analysis
Source: Sci Rep. 2026 Apr 25;16:19164. doi: 10.1038/s41598-026-47007-6 (PMC13279806; doi:10.1038/s41598-026-47007-6)
Supplement: Supplementary file 2 — Supplementary Material 2 [file 41598_2026_47007_MOESM2_ESM.docx]

| **Supplemental Table 1.** Definition of injuries and procedures | | | |
| --- | --- | --- | --- |
|  | **ICD-9 Code*** | **ICD-10 Code*** | **AIS code** |
| Grade III Kidney Injury |  |  | 541614, 541624 |
| Grade IV Kidney Injury |  |  | 541626, 541640 |
| Renal Artery Injury | 902.41 | S35.401, S35.402, S35.403,  S35.411, S35.412, S35.413,  S35.491, S35.492, S35.493 |  |
| Renal Vein Injury | 902.42 | S35.404, S35.405, S35.406,  S35.414, S35.415, S35.416,  S35.494, S35.495, S35.496 |  |
| Laparotomy | 54.1 | 0DJ00ZZ, 0DJ60ZZ,  0DJD0ZZ, 0DJU0ZZ,  0DJW0ZZ, 0WJG0ZZ,  0WJJ0ZZ, 0WJP0ZZ,  0WJR0ZZ |  |
| Partial Nephrectomy | 55.4 | 0TB00ZZ, 0TB03ZZ,  0TB04ZZ, 0TB07ZZ,  0TB08ZZ, 0TB10ZZ,  0TB13ZZ, 0TB14ZZ,  0TB17ZZ, 0TB18ZZ,  0TB30ZZ, 0TB33ZZ,  0TB34ZZ, 0TB37ZZ,  0TB38ZZ, 0TB40ZZ,  0TB43ZZ, 0TB44ZZ,  0TB47ZZ, 0TB48ZZ,  0TT30ZZ, 0TT34ZZ,  0TT37ZZ, 0TT38ZZ,  0TT40ZZ, 0TT44ZZ,  0TT47ZZ, 0TT48ZZ |  |
| Total Nephrectomy | 55.5 | 0TT00ZZ, 0TT04ZZ,  0TT10ZZ, 0TT14ZZ |  |
| Surgery on the  gastrointestinal system | 42, 43, 44,  45, 46, 47,  48, 49 | 0D |  |
| Surgery on the urinary system  (excluding codes for nephrectomy) | 55, 56, 57,  58, 59 | 0T |  |
| Surgery on the  hepatobiliary system or pancreas | 50, 51, 52 | 0F |  |
| **ICD codes listed represent the base code and all associated subcodes* | | | |

| **Supplemental Table 2.** Association between nephrectomy and outcomes in patients without hypotension with a grade III kidney injury due to penetrating trauma | | | |
| --- | --- | --- | --- |
| **Outcome** | **Renal Salvage** | **Nephrectomy**  **PR (95% CI)** | **P-Value** |
| In-hospital mortality | Reference | 1.52 (0.89-2.58) | 0.124 |
| Any complication | Reference | 1.24 (1.01-1.52) | 0.040 |
| Any complication, excluding acute kidney injury | Reference | 1.21 (0.97-1.50) | 0.086 |
| Post-complication mortality | Reference | 1.41 (0.69-2.87) | 0.346 |
| ICU admission | Reference | 1.09 (1.03-1.16) | 0.004 |
|  |  | **Change in median (95% CI)** | **P-Value** |
| Length of stay | Reference | 1.70 (0.02-3.38) | 0.048 |
| PRs are calculated using Poisson regression models with robust standard errors. Median length of stay is calculated using a quantile regression model. Missing values were managed using multiple imputation by chained equations. All analyses were adjusted for age, sex, race, highest abbreviated injury scale in each region, vitals on admission, presence of renal artery or renal vein injury, comorbidities, advanced directives limiting care, surgery on the gastrointestinal system, surgery on the hepatobiliary system or pancreas, surgery on the urinary system (excluding nephrectomy), units of packed red blood cells transfused within 4 hours, mechanism of injury, trauma center level, year of admission.  *PR, Prevalence Ratio; CI, Confidence Interval* | | | |

| **Supplemental Table 3.** Association between nephrectomy and outcomes in patients without hypotension with a grade IV kidney injury due to penetrating trauma | | | |
| --- | --- | --- | --- |
| **Outcome** | **Renal Salvage** | **Nephrectomy**  **PR (95% CI)** | **P-Value** |
| In-hospital mortality | Reference | 0.94 (0.72-1.23) | 0.661 |
| Any complication | Reference | 0.98 (0.86-1.12) | 0.801 |
| Any complication, excluding acute kidney injury | Reference | 0.98 (0.86-1.12) | 0.775 |
| Post-complication mortality | Reference | 0.65 (0.41-1.01) | 0.056 |
| ICU admission | Reference | 1.03 (0.99-1.07) | 0.121 |
|  |  | **Change in median (95% CI)** | **P-Value** |
| Length of stay | Reference | 0.35 (-0.82-1.52) | 0.558 |
| PRs are calculated using Poisson regression models with robust standard errors. Median length of stay is calculated using a quantile regression model. Missing values were managed using multiple imputation by chained equations. All analyses were adjusted for age, sex, race, highest abbreviated injury scale in each region, vitals on admission, presence of renal artery or renal vein injury, comorbidities, advanced directives limiting care, surgery on the gastrointestinal system, surgery on the hepatobiliary system or pancreas, surgery on the urinary system (excluding nephrectomy), units of packed red blood cells transfused within 4 hours, mechanism of injury, trauma center level, year of admission.  *PR, Prevalence Ratio; CI, Confidence Interval* | | | |
